# LC3, an autophagosome marker, is expressed on oligodendrocytes in Nasu-Hakola disease brains

**DOI:** 10.1186/1750-1172-9-68

**Published:** 2014-05-01

**Authors:** Jun-ichi Satoh, Nobutaka Motohashi, Yoshihiro Kino, Tsuyoshi Ishida, Saburo Yagishita, Kenji Jinnai, Nobutaka Arai, Kiyotaka Nakamagoe, Akira Tamaoka, Yuko Saito, Kunimasa Arima

**Affiliations:** 1 Department of Bioinformatics and Molecular Neuropathology, Meiji Pharmaceutical University, Tokyo, Japan; 2 Department of Psychiatry, University of Yamanashi, Faculty of Medicine, Yamanashi, Japan; 3 Department of Pathology and Laboratory Medicine, Kohnodai Hospital, National Center for Global Health and Medicine, Chiba, Japan; 4 Department of Pathology, Kanagawa Rehabilitation Center, Kanagawa, Japan; 5 Department of Neurology, NHO Hyogo-Chuo Hospital, Hyogo, Japan; 6 Brain Pathology Research Center, Tokyo Metropolitan Institute of Medical Science, Tokyo, Japan; 7 Department of Neurology, Institute of Clinical Medicine, University of Tsukuba, Ibaraki, Japan; 8 Department of Laboratory Medicine, National Center Hospital, NCNP, Tokyo, Japan; 9 Department of Psychiatry, National Center Hospital, NCNP, Tokyo, Japan

**Keywords:** Autophagy, LC3, Leukoencephalopathy, Nasu-Hakola disease, Oligodendrocytes

## Abstract

**Background:**

Nasu-Hakola disease (NHD) is a rare autosomal recessive disorder characterized by sclerosing leukoencephalopathy and multifocal bone cysts, caused by a loss-of-function mutation of either DAP12 or TREM2. TREM2 and DAP12 constitute a receptor/adaptor signaling complex expressed exclusively on osteoclasts, dendritic cells, macrophages, and microglia. Neuropathologically, NHD exhibits profound loss of myelin and accumulation of axonal spheroids, accompanied by intense gliosis accentuated in the white matter of the frontal and temporal lobes. At present, the molecular mechanism responsible for development of leukoencephalopathy in NHD brains remains totally unknown.

**Methods:**

By immunohistochemistry, we studied the expression of microtubule-associated protein 1 light chain 3 (LC3), an autophagosome marker, in 5 NHD and 12 control brains.

**Results:**

In all NHD brains, Nogo-A-positive, CNPase-positive oligodendrocytes surviving in the non-demyelinated white matter intensely expressed LC3. They also expressed ubiquitin, ubiquilin-1, and histone deacetylase 6 (HDAC6) but did not express Beclin 1 or sequestosome 1 (p62). Substantial numbers of axonal spheroids were also labeled with LC3 in NHD brains. In contrast, none of oligodendrocytes expressed LC3 in control brains. Furthermore, surviving oligodendrocytes located at the demyelinated lesion edge of multiple sclerosis (MS) did not express LC3, whereas infiltrating Iba1-positive macrophages and microglia intensely expressed LC3 in MS lesions.

**Conclusions:**

These results propose a novel hypothesis that aberrant regulation of autophagy might induce oligodendrogliopathy causative of leukoencephalopathy in NHD brains.

## Background

Nasu-Hakola disease (NHD), also designated polycystic lipomembranous osteodysplasia with sclerosing leukoencephalopathy (PLOSL; OMIM 221770), is a rare autosomal recessive disorder, characterized by progressive presenile dementia and formation of multifocal bone cysts [1,2]. Although NHD patients are clustered in Japan and Finland, approximately 200 NHD cases are presently reported worldwide (http://www.orpha.net). Clinically, the patients show pathological bone fractures during the third decade of life, and a frontal lobe syndrome, such as loss of judgment and social inhibitions during the fourth decade of life, followed by progressive dementia and death until the fifth decade of life [3]. Pathologically, NHD brains exhibit extensive demyelination with sparing of subcortical U-fibers, accumulation of axonal spheroids, and intense astrogliosis predominantly in the white matter of frontal and temporal lobes and the basal ganglia [4]. Genetically, NHD is caused by the set of heterogeneous mutations located in one of the two genes, DNAX-activation protein 12 (*DAP12*), alternatively named TYRO protein tyrosine kinase-binding protein (*TYROBP*) on chromosome 19q13.1 or triggering receptor expressed on myeloid cells 2 (*TREM2*) on chromosome 6p21.1 [5-7]. Previous studies identified 7 different mutations in the *TYROBP* gene and 11 distinct mutations in the *TREM2* gene in NHD patients. The presence of multiple bone cysts, basal ganglia calcification, and genetic mutations of *TYROBP* or *TREM2* in a pattern of autosomal recessive inheritance could differentiate NHD from hereditary diffuse leukoencephalopathy with spheroids (HDLS; OMIM 221820), a rare autosomal dominant disorder presenting with clinicopathological similarities to NHD, which is caused by genetic mutations in the colony-stimulating factor 1 receptor (CSF1R) gene [8].

TREM2, expressed exclusively on myeloid cells, such as osteoclasts, dendritic cells, macrophages, and microglia, acts as a receptor for as yet unidentified ligands. TREM2 constitutes a signaling complex with an adaptor molecule DAP12, leading to phosphorylation and activation of the downstream kinase named spleen tyrosine kinase (Syk), following the receptor engagement [9]. Syk transduces a wide range of downstream signals involved in activation of phosphatidylinositol-3 kinase (PI3K), phospholipase C (PLC), protein kinase C (PKC), and mitogen-activated protein kinase (MAPK) [10].

Increasing evidence indicated that a defect in microglial TREM2/DAP12 function plays a central role in the pathogenesis of NHD [11]. However, at present, the molecular mechanism responsible for development of leukoencephalopathy in NHD brains remains totally unknown. DAP12-knockout mice develop osteopetrosis, thalamic hypomyelination, and synaptic degeneration [12], being phenotypically different from osteolytic lesions and sudanophilic leukoencephalopathy found in NHD patients. Several studies showed that oligodendrocytes, along with microglia, express DAP12 [12,13]. However, follow-up studies could not verify oligodendroglial expression of DAP12 [14]. The synaptic function is also altered in DAP12 loss-of-function (K∆75) mice, attributable to reduced expression of AMPA receptor GluR2 subunit and neurotrophin receptor TrkB [15]. Furthermore, the total number of microglia is greatly reduced in the brain of DAP12-deficient and loss-of-function mice [16,17]. These observations suggest that DAP12 signaling pathway plays a key role in development of microglia and maturation of synapses. Knockdown of TREM2 on cultured mouse microglia inhibits phagocytosis of apoptotic neurons, and stimulates production of proinflammatory cytokines, such as TNFα and IL-1β, suggesting that TREM2 plays a key role in the clearance of dying neural cells by microglia to resolve damage-induced inflammation [18]. In contrast to the suggested role of microglial TREM2 in the pathogenesis of NHD, we recently found that TREM2 is not expressed constitutively on human microglia, and Iba1-positive microglia are well preserved in the brains of NHD patients with DAP12 mutations [19].

Macroautophagy, hereafter called as autophagy, constitutes a lysosome-mediated degradation process that controls the quality of cytoplasmic components and organelles [20,21]. The process of autophagy involves the complex molecular machinery, composed of more than 30 autophagy-related (Atg) proteins and 50 lysosomal hydrolases. Autophagy is initiated by the formation of double-membrane-bound vesicles named autophagosomes that sequester cytoplasmic material in a non-degenerative compartment, followed by fusion with lysosomes, leading to degradation of the autophagic contents. They provide recycling pools of nutrients and membranes, being essential for maintenance of the cellular homeostasis and renovation. When the cells are exposed to protein-damaging insults, autophagy plays a key role in eliminating protein aggregates and damaged organelles, both of which are resistant to degradation by the ubiquitin-proteasome system (UPS) [20,21]. Mice defective in autophagy show severe neurodegeneration accompanied by an accumulation of ubiquitinated protein aggregates [22]. Furthermore, abnormal regulation of autophagy plays a central role in the pathogenesis of human neurodegenerative diseases, such as Alzheimer’s disease (AD) and Parkinson’s disease (PD), accompanied by neuronal accumulation of insoluble protein aggregates [23].

Because TREM2 serves as a phagocytic receptor of apoptotic neurons [18,24], and the efficient clearance of dead cells requires microtubule-associated protein 1 light chain 3A (LC3)-associated phagocytosis [25], we attempted to study the expression of LC3 in NHD brains by immunohistochemistry. Unexpectedly, we found that LC3 expression is enhanced on oligodendrocytes in NHD brains but not in control brains.

## Methods

### Human brain tissues

Formalin-fixed paraffin-embedded brain tissues of the cerebral cortex, the hippocampus, and the basal ganglia derived from NHD and non-NHD cases were obtained from the Research Resource Network (RRN), Japan. Written informed consent was taken in all the cases at autopsy, following the regulation of the institutional ethics committees. The present study includes five NHD patients, composed of a 42-year-old man (NHD1), a 48-year-old woman (NHD2), a 44-year-old man (NHD3), a 32-year-old woman (NHD4), and a 38-year-old man (NHD5), four neuropsychiatric disease controls affected with myotonic dystrophy (MD), composed of a 68-year-old man (MD1), a 61-year-old man (MD2), a 60-year-old man (MD3), and a 53-year-old woman (MD4), four demyelinating disease controls affected with chronic progressive multiple sclerosis (MS), composed of a 29-year-old woman (MS1), a 40-year-old woman (MS2), a 43-year-old woman (MS3), and a 33-year-old man (MS4), and four subjects who died of non-neurological causes (NC), composed of a 63-year-old man who died of prostate cancer and acute myocardial infarction (NC1), a 67-year-old man who died of dissecting aortic aneurysm (NC2), a 57-year-old man who died of alcoholic liver cirrhosis (NC3), and a 61-year-old man who died of rheumatoid arthritis with interstitial pneumonia (NC4). The homozygous mutation of a single base deletion of 141G (141delG) in exon 3 of DAP12 was identified in NHD1, NHD2, and NHD5 [19,26], while the genetic analysis was not performed in NHD3 [27] or NHD4 [28].

### Immunohistochemistry

After deparaffination, tissue sections were heated in 10 mM citrate sodium buffer, pH 6.0 or 9.0 by autoclave at 110°C for 15 min in a temperature-controlled pressure chamber (Biocare Medical, Concord, CA, USA). They were treated at room temperature (RT) for 15 min with 3% hydrogen peroxide-containing methanol to block the endogenous peroxidase activity. They were then incubated with phosphate-buffered saline (PBS) containing 10% normal goat or rabbit serum at RT for 15 min to block non-specific staining, followed by incubation in a moist chamber at 4°C overnight with the primary antibodies listed in Table 1. We selected Nogo-A as the most reliable marker highly specific for oligodendrocytes in human brain tissue sections, as reported previously [29]. After washing with PBS, the tissue sections were incubated at RT for 30 min with horseradish peroxidase (HRP)-conjugated secondary antibodies (Nichirei, Tokyo, Japan), followed by incubation with diaminobenzidine tetrahydrochloride (DAB) substrate (Vector, Burlingame, CA, USA). They were processed for a counterstain with hematoxylin. Negative controls underwent all the steps except for exposure to primary antibody.

**Table 1:** Primary antibodies utilized for immunohistochemistry in the present

**Antibody**	**Supplier**	**Code (ID)**	**Origin**	**Antigen**	**Concentration**
LC3	MBL	PM036	rabbit	recombinant human LC3B spanning amino acid residues 1-120 aa	diluted at 1: 5000
BECN1	AnaSpec	54229	rabbit	a peptide mapping near the N-terminus of human Beclin-1	0.2 μg/ml
NBR1	ProteinTech	16004-1-AP	rabbit	recombinant human NBR1-6xHis fusion protein	0.26 μg/ml
HDAC6	Santa Cruz Biotechnology	sc-11420	rabbit	a peptide spanning amino acid residues 916-1215 of human HDAC6	0.8 μg/ml
p62/SQSTM1	BD Bioscience	610832	mouse	a peptide spanning amino acid residues 257-437 of human p62	1 μg/ml
Ubiquitin	Dako	Z0458	rabbit	ubiquitin isolated from bovine erythrocytes	0.25 μg/ml
UBQLN1	Santa Cruz Biotechnology	sc-14652	goat	a peptide mapping within an internal region of human ubiquilin-1	1 μg/ml
Nogo-A	Santa Cruz Biotechnology	H-300	rabbit	a peptide mapping amino acids 700-1000 of human Nogo-A	0.1 μg/ml
MBP	Dako	N1564	rabbit	MBP purified from human brain	prediluted
CNPase	Sigma	11-5B	mouse	purified human CNPase	ascites fluid 1:500
Iba1	Wako	019-19741	rabbit	a synthetic peptide corresponding to the C-terminus of Iba1	0.5 μg/ml
GFAP	Dako	N1506	rabbit	GFAP purified from bovine spinal cord	prediluted
NF	Nichirei	412551 (2 F11)	mouse	NF purified from human brain	prediluted
Cleaved CASP3	Cell Signaling Technology	#9661 (Asp175)	rabbit	a peptide mapping amino-terminal residues adjacent to Asp175 of human caspase-3	1:100

*Abbreviations:* LC3, microtubule-associated protein 1 light chain 3; BECN1, Beclin 1; NBR1, neighbor of BRCA1 gene 1; HDAC6, histone deacetylase 6; SQSTM1, sequestosome 1; UBQLN1, ubiquilin-1; MBP, myelin basic protein; CNPase, 2′,3′-cyclic nucleotide 3′ phosphodiesterase; GFAP, glial fibrillary acidic protein; NF, neurofilament protein; and CASP3, caspase-3.

### Western blot analysis

To prepare total protein extract, the cells were homogenized in the mammalian protein extraction reagent (M-PER; Thermo Scientific, Rockford, IL, USA) supplemented with a cocktail of protease inhibitors (Sigma, St. Louis, MO, USA). The protein extract was centrifuged at 12,000 rpm for 5 min at RT, separated on a 15% SDS-PAGE gel, and transferred onto nitrocellulose membranes. They were labeled at RT overnight with rabbit anti-LC3 antibody (PM036; MBL International, Woburn, MA, USA) that react with MAP1LC3A/B/C or goat anti-heat shock protein HSP60 antibody (sc-1052, N-20; Santa Cruz Biotechnology, Santa Cruz, CA, USA) to standardize protein loading. Then, the membranes were incubated at RT for 60 min with HRP-conjugated anti-rabbit or anti-goat IgG (Santa Cruz Biotechnology). The specific reaction was visualized by exposing the membranes to a chemiluminescent substrate (Thermo Scientific).

## Results

### Oligodendrocytes surviving in the non-demyelinated white matter of NHD brains intensely expressed LC3 immunoreactivity

**Figure 1:**
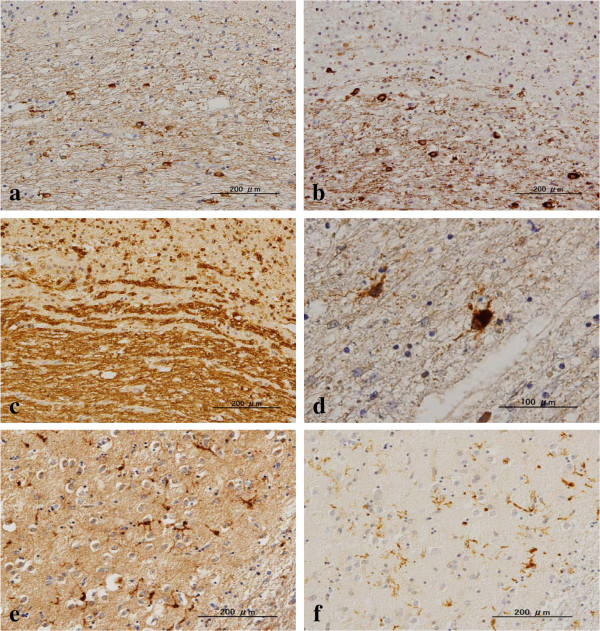
**Surviving oligodendrocytes express LC3 in NHD brains.** The serial brain sections of NHD cases were processed for immunohistochemistry. The panels **(a-f)** represent **(a)** the periventricular white matter, LC3, **(b)** the same field as **(a)**, Nogo-A, **(c)** the same field as **(a)**, MBP, the upper half indicates demyelinated lesions, **(d)** the frontal white matter, LC3, **(e)** the basal ganglia, LC3, and **(f)** the same field as **(e)**, Iba1.

**Figure 2:**
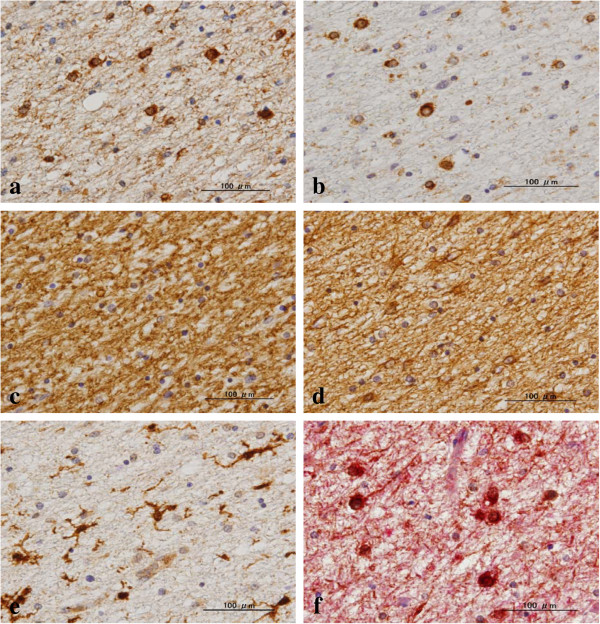
**Surviving oligodendrocytes express LC3 in NHD brains.** The serial brain sections of NHD cases were processed for immunohistochemistry. The panels **(a-f)** represent the identical field of the frontal white matter labeled with **(a)** LC3, **(b)** Nogo-A, **(c)** MBP, **(d)** GFAP, **(e)** Iba1, and **(f)** CNPase (red) and LC3 (brown).

**Figure 3:**
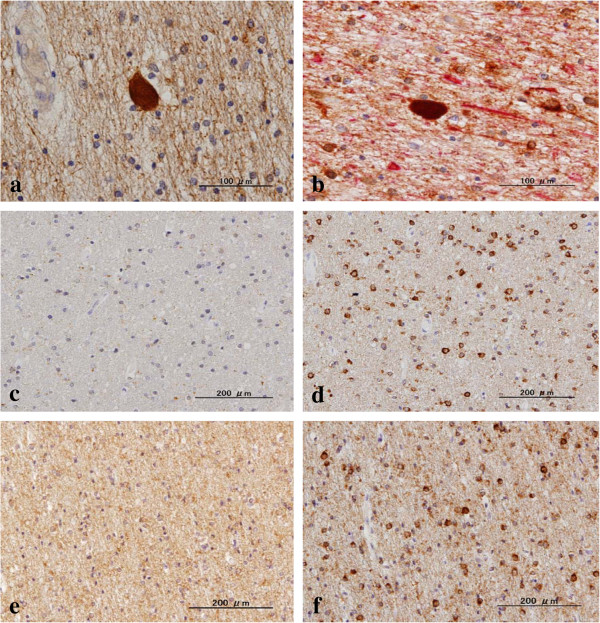
**Oligodendrocytes do not express LC3 in control brains.** The serial brain sections of NHD, myotonic dystrophy (MD), and non-neurological control (NC) cases were processed for immunohistochemistry. The panels **(a-f)** represent **(a)** NHD, the basal ganglia, LC3, **(b)** NHD, the frontal white matter, neurofilament protein (red) and LC3 (brown), **(c)** NC, the frontal white matter, LC3, **(d)** the same field as **(c)**, Nogo-A, **(e)** MD, the frontal white matter, LC3, and **(f)** the same field as **(e)**, Nogo-A.

First, we validated the specificity of anti-LC3 antibody PM036 by western blot analysis of total protein extracted from mouse oligodendrocyte-type 2 astrocyte (O2A) progenitor cells termed OS3 [30], following a 48-hour exposure to rapamycin, a potent inducer of autophagy. This antibody reacted with both LC3-I, the soluble cytosolic form and LC3-II, the autophagy-inducible phosphatidylethanolamine (PE)-conjugated form (Additional file 1: Figure S1a, b, lanes 1, 2). Then, we studied the expression of LC3 in the serial brain sections of five NHD, four MD, and four NC cases by immunohistochemistry using the PM036 antibody. In all cases examined, substantial populations of cortical neurons constitutively expressed LC3 in the cytoplasm at varying intensities. Notably, in all five NHD brains, Nogo-A-positive, cleaved caspase-3 (CASP3)-negative (non-apoptotic) oligodendrocytes surviving in the MBP-positive (non-demyelinated) white matter intensely expressed LC3 with the location in the cytoplasm (Figure 1a-c, Figure 2a-c; Additional file 2: Figure S2a, b). Some LC3-immunolabeled oligodendrocytes showed a morphological feature of swollen cytoplasm (Figure 1d). In contrast, extensively demyelinated white matter, almost totally devoid of oligodendrocytes, was not labeled with anti-LC3 antibody (Figure 1a-c, upper half). Double labeling verified that LC3-expressing cells accumulated in the non-demyelinated white matter of NHD brains coexpressed 2’,3’-cyclic nucleotide 3′ phosphodiesterase (CNPase), a cell type-specific marker of oligodendrocytes (Figure 2f). The distribution of LC3 immunoreactivity (Figure 2a) was well consistent with the staining pattern of Nogo-A (Figure 2b) but not of GFAP (Figure 2d) or Iba1 (Figure 2e), although some populations of ramified microglia, accumulating macrophages, and reactive astrocytes expressed intensely LC3 in NHD brains (Figure 1e, f). In NHD brains, substantial numbers of axonal spheroids were also labeled with LC3, along with neurofilament (Figure 3a, b). In contrast, we found no LC3-expressing oligodendrocytes in the white matter of control brains, including NC and MD cases (Figure 3c-f).

### LC3-positive oligodendrocytes did not express p62 or beclin 1 in NHD brains

**Figure 4:**
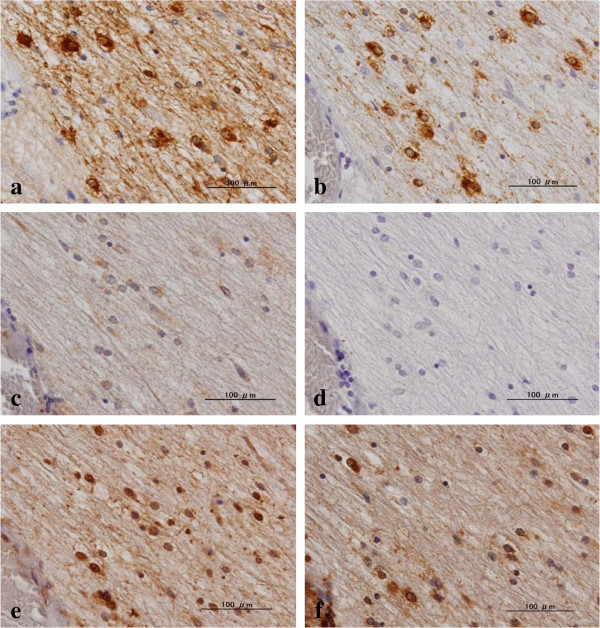
**Surviving oligodendrocytes do not express Beclin 1 or p62 in NHD brains.** The serial brain sections of NHD cases were processed for immunohistochemistry. The panels **(a-f)** represent the identical field of the frontal white matter labeled with **(a)** LC3, **(b)** Nogo-A, **(c)** Beclin 1, **(d)** p62, **(e)** ubiquitin, and **(f)** UBQLN1.

Next, we studied the expression of a panel of autophagy regulators, such as Beclin 1 (ATG6), p62 or NBR1 in NHD brains. Nogo-A-positive LC3-positive oligodendrocytes did not express either Beclin 1 or p62 (Figure 4a-d), although a subpopulation of LC3-positive oligodendrocytes fairly weakly expressed neighbor of BRCA1 gene 1 (NBR1) (Additional file 2: Figure S2c). In contrast, the majority of LC3-positive oligodendrocytes intensely or moderately expressed histone deacetylase 6 (HDAC6) in the cytoplasm (Additional file 2: Figure S2d). Furthermore, many LC3-positive oligodendrocytes moderately expressed both ubiquitin and ubiquilin-1 (UBQLN1) (Figure 4e, f). These observations suggest that the expression of UPS components is upregulated in surviving oligodendrocytes distributed in the non-demyelinating white matter of NHD brains.

### Oligodendrocytes surviving at the demyelinated lesion edge in MS brains did not express LC3

**Figure 5:**
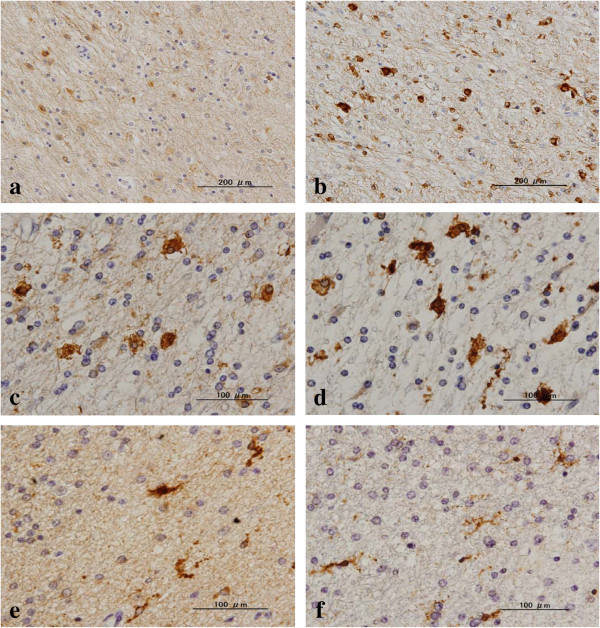
**Macrophages and microglia but not oligodendrocytes express LC3 in MS brains.** The serial brain sections of multiple sclerosis (MS) cases were processed for immunohistochemistry. The panels **(a-f)** represent **(a)** the edge of demyelinated lesions in the parietal white matter, LC3, **(b)** the same field of **(a)**, Nogo-A, **(c)** the edge of demyelinated lesions in the frontal white matter, LC3, **(d)** the same field as **(c)**, Iba1, **(e)** the edge of demyelinated lesions in the frontal white matter, LC3, and **(f)** the same field as **(e)**, Iba1.

Finally, to investigate whether oligodendroglial LC3 expression represents a general biological process during demyelination, we studied the expression of LC3 in the cerebral white matter of the brains derived from four MS patients. At the edge of chronic active demyelinated lesions, surviving Nogo-A-positive oligodendrocytes did not express LC3 (Figure 5a, b), whereas infiltrating Iba1-positive macrophages and microglia intensely expressed LC3 in MS lesions (Figure 5e-f). Furthermore, none of Nogo-A-positive oligodendrocytes expressed LC3 in earlier lesions as well as normal-appearing white matter (NAWM) of MS brains (Additional file 3: Figure S3a, b). These observations suggest that oligodendroglial expression of LC3 is not unique to demyelinating events.

## Discussion

Here, we found that oligodendrocytes surviving in the non-demyelinated white matter of NHD brains but not in the white matter of control brains intensely expressed LC3, the most reliable *in vivo* marker of autophagosomes. LC3-positive oligodendrocytes also expressed ubiquitin, ubiqulin-1 and HDAC6, whereas they marginally expressed NBR1 and did not express Beclin 1 or p62. Furthermore, a subset of axonal spheroids expressed LC3 in NHD brains. Since oligodendrocytes support axonal function by continuously supplying energy metabolites to axons [31], a functional relationship might exist between LC3-expressing oligodendrocytes and axonal spheroids. In contrast, surviving oligodendrocytes located at the demyelinated lesion edge of MS did not express LC3, indicating that different molecular mechanisms might be involved in demyelinating processes between NHD and MS. It is well known that demyelinated lesions in MS brains have a well-demarcated border but the lesions are ill-defined and diffuse in the white matter of NHD brains [4]. Cortical demyelination is common in MS brains [32], while the architecture of the cerebral cortex is well preserved in NHD brains [33]. Demyelinated lesions of MS are often accompanied by perivascular infiltration of numerous T lymphocytes, while NHD brain lesions contain a limited number of CD3-positive T cells [19], supporting the general view that MS is a T cell-mediated autoimmune disease affecting the central nervous system white matter, whereas autoimmune mechanisms are unlikely to play a central role in the pathogenesis of NHD. Previously, we found that the levels of expression of a guanine nucleotide exchanger for Rap termed RAPGEF4, which plays a role in the inhibition of autophagy [34], are greatly reduced in NHD brains [35]. All of these observations suggest the hypothesis that aberrant regulation of autophagy might induce oligodendrogliopathy causative of leukoencephalopathy in NHD brains.

Autophagy is mediated by the molecular machinery that involves numerous regulatory proteins [20,21]. Recently, more than 400 interacting proteins that constitute the basal autophagy network have been identified in human cells, representing the extreme complexity of autophagy [36]. LC3 (ATG8), synthesized as a precursor form, is cleaved at its C-terminus by the cysteine protease ATG4B, which generates the cytosolic isoform termed LC3-I [20]. During the phagophore elongation, LC3-I is conjugated to PE via a reaction that involves ATG7 and ATG3 to form LC3-II that is specifically targeted to the elongating autophagosomal membranes. Following the fusion of autophagosomes with lysosomes, LC3-II located on the cytoplasmic face of autolysosomes is delipidated by ATG4 and processed for recycling, while LC3-II on the internal surface of autophagosomes is processed for degradation by lysosomal enzymes of autolysosomes [20]. All currently available anti-LC3 antibodies, including the PM036 antibody utilized in the present study, recognize both LC3-I and LC3-II. When autophagosomes are accumulated in the cell extremely in number due to excessive induction or reduced completion of autophagy, LC3 intensities are elevated chiefly by an increase in LC3-II expression on autophagosomal membranes.

Under physiological conditions, UPS mainly regulates degradation of short-lived polyubiquitinated proteins, while autophagy predominantly degrades long-lived proteins having higher-ordered structures inaccessible to the narrow pore of the barrel structure of the proteasome, although functionally redundant interactions exist between the two systems [37]. A battery of autophagic receptors/adaptors that connect the UPS and autophagy, such as p62, NBR1, UBQLN1, optineurin (OPTN), and HDAC6, recognize ubiquitinated target proteins and promote their degradation by autophagy [38]. Importantly, p62, NBR1, UBQLN1, and OPTN have a capacity to bind directly to LC3 [39,40]. We found that LC3-positive oligodendrocytes intensely express UBQLN1 and HDAC6, both of which play a pivotal role in the aggresome formation [41,42]. In contrast, LC3-positive oligodendrocytes did not much express either p62 or NBR1. It is worthy to note that p62 knockout mice do not show a defect in bulk autophagy, suggesting that p62 is primarily dispensable for the clearance of autophagic substrates [43].

Under stressful conditions, autophagy serves as a protective mechanism for the cell to prevent the accumulation of cytotoxic protein aggregates and damaged organelles [20,21]. Actually, increased levels of autophagy promote survival of oligodendrocytes in a myelin-deficient rat [44]. However, uncontrolled activation of autophagy often induces cell death. During ischemia-reperfusion injury of the myocardium, early activation of autophagy upon ischemia is protective, while delayed and robust activation of autophagy during reperfusion is detrimental for cell survival [45]. Furthermore, a tight linkage is found between autophagy and apoptosis. The autophagy inhibitor 3-methyladenine (3-MA) inhibits apoptotic cell death of TNFα-treated T lymphoblastic leukemia cells and NGF-deprived sympathetic neurons [46,47]. The prototype anti-apoptotic regulator Bcl-2 inhibits starvation-induced autophagy by directly interacting with Beclin 1 [48].

At present, the precise mechanism remains unknown how microglial dysfunction termed microgliopathy caused by the genetic defect of DAP12 or TREM2 induces oligodendrogliopathy characterized by enhanced LC3 expression on oligodendrocytes in NHD brains. It is possible that microglia persistently deregulated in NHD brains produce excessive amounts of reactive oxygen species (ROS) that potentially activate autophagy in oligodendrocytes. In turn, autophagy itself controls inflammation through regulatory interactions with innate immune signaling pathways [40]. By gene expression profiling, we recently identified 324 DEGs expressed in frozen brain tissues of a NHD patient with a splicing mutation of TREM2 [35]. Among them, the set of 136 genes involved in inflammatory response and immune cell trafficking are upregulated, while the set of 188 genes including a battery of GABA receptor subunits and synaptic proteins are downregulated in NHD brains. These observations suggested that both neuroinflammatory and neurodegenerative events proceed concurrently in NHD brains. Notably, the expression of a set of macrophage/microglia markers, such as CD163, MSR1, and CD68, is greatly elevated in NHD brains [35].

Upregulation of LC3 is attributable to increased autophagic flux or decreased autophagic substrate clearance, or both. Diverse stress-inducing stimuli, including exposure to ROS and deprivation of nutrients, growth factors, or adenosine triphosphate (ATP), all turns on autophagy by inhibiting the mammalian target of rapamycin complex 1 (mTORC1) [20]. Notably, rapamycin, a relatively selective inhibitor of mTORC1, ameliorates neurodegeneration in mouse models of AD, PD, and frontotemporal lobar degeneration (FTLD), where neuronal cell death is attributable to a defect in autophagy [49,50]. On the contrary, activation of mTORC1 and mTORC2 is pivotal for oligodendrocytes differentiation at the stage of transition from the late progenitors to immature oligodendrocytes [51]. All of these observations suggest that delicate regulation of cellular autophagy levels plays a decisive role in neural cell survival or cell death.

## Conclusions

We for the first time found that LC3 is expressed on surviving oligodendrocytes in the non-demyelinated white matter of NHD brains but not in the white matter of control brains. These observations propose a novel hypothesis that aberrant regulation of autophagy might induce oligodendrogliopathy causative of leukoencephalopathy in NHD brains.

## Abbreviations

AD: Alzheimer’s disease; CNPase: 2’,3’-cyclic nucleotide 3′ phosphodiesterase; DAP12: DNAX-activation protein 12; HDAC6: Histone deacetylase 6; LC3: microtubule-associated protein 1 light chain 3; MD: Myotonic dystrophy; MS: Multiple sclerosis; NBR1: Neighbor of BRCA1 gene 1; NHD: Nasu-Hakola disease; PD: Parkinson’s disease; TREM2: Triggering receptor expressed on myeloid cells 2; UPS: Ubiquitin-proteasome system.

## Competing interests

The authors declare that they have no competing interests.

## Authors’ contributions

JS and YK performed immunohistochemical analysis. JS drafted the manuscript. NM, SY, KJ, NA, KN, and AT provided NHD brain tissues. KA, YS, and TI validated the pathological diagnosis of all autopsied brains. All authors read and approved the final manuscript.

## Supplementary Material

Additional file 1: Figure S1 Validation of the specificity of anti-LC3 antibody. Total protein extracted from oligodendrocyte-type 2 astrocyte (O2A) progenitor cells named OS3 was processed for western blot with (a) anti-LC3 antibody PM036 and relabeled with (b) anti-HSP60 antibody for standardization of protein loading. The lanes (1, 2) indicate a 48 hour-treatment of OS3 cells with (1) the equal v/v% concentration of dimethyl sulfoxide (DMSO) or (2) 1 μM rapamycin. Click here for file

Additional file 2: Figure S2 Surviving oligodendrocytes express HDAC6 in NHD brains. The serial brain sections of NHD cases were processed for immunohistochemistry. The panels (a-d) represent (a) the perivascular white matter, LC3, (b) the same field as (a), cleaved CASP3, (c) the same filed as (a), NBR1, and (d) the same field as (a), HDAC6 with a close-up view in inset. Click here for file

Additional file 3: Figure S3 Oligodendrocytes do not express LC3 in early lesions of MS brains. The serial brain sections of MS cases were processed for immunohistochemistry. The panels (a, b) represent (a) an early lesion in the frontal white matter, LC3, some macrophages are positive, and (b) the same field as (a), Nogo-A. Click here for file
