# Supplementary figures and images for: LC3, an autophagosome marker, is expressed on oligodendrocytes in Nasu-Hakola disease brains
Source: Orphanet J Rare Dis. 2014 May 1;9:68. doi: 10.1186/1750-1172-9-68 (PMC4022378; doi:10.1186/1750-1172-9-68)

## Slide 1
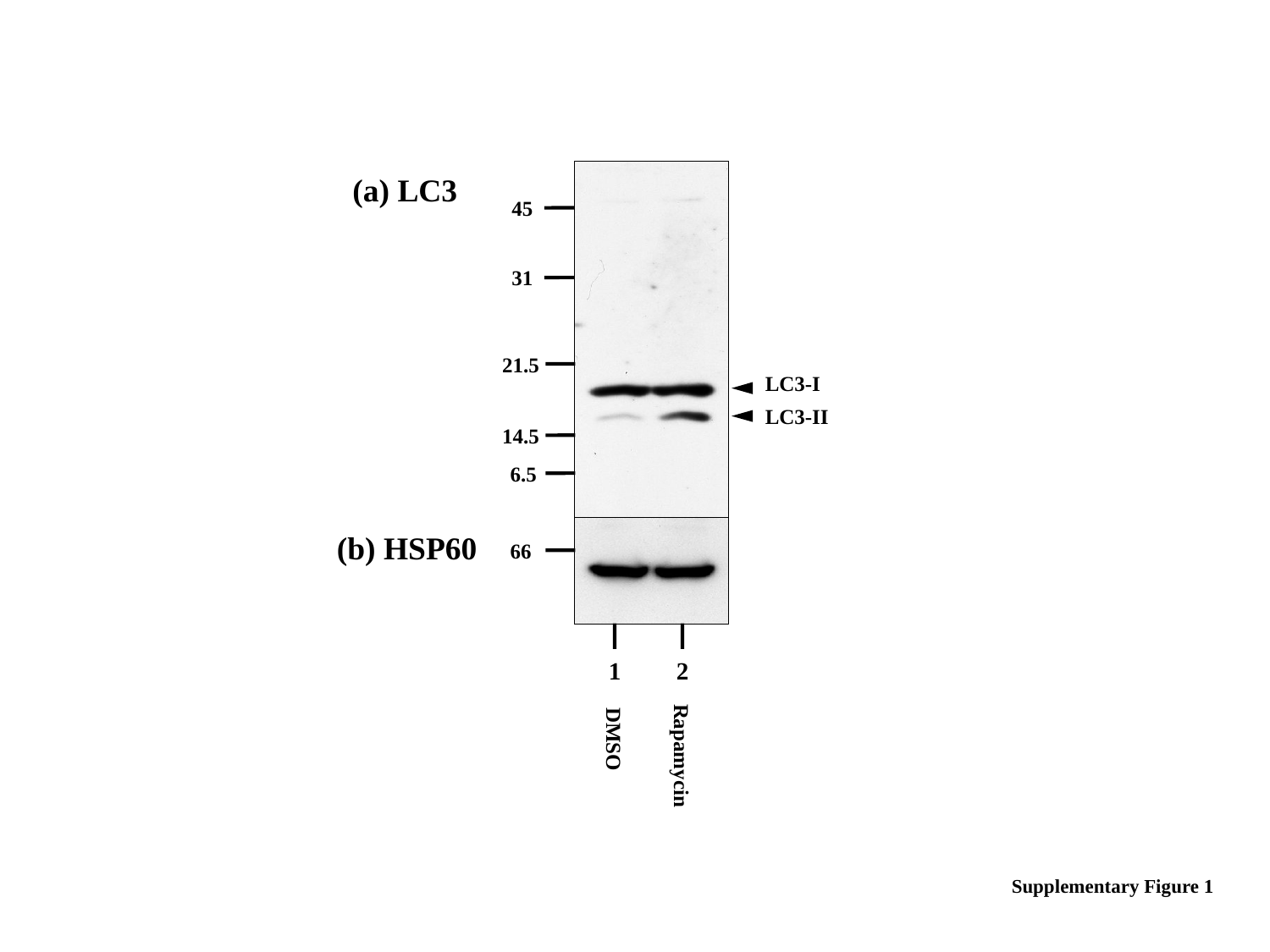

(a) LC3
45
31
21.5
LC3-I
LC3-II
14.5
6.5
(b) HSP60
66
1
2
DMSO
Rapamycin
Supplementary Figure 1

Supplement: Additional file 1: Figure S1 — Validation of the specificity of anti-LC3 antibody. Total protein extracted from oligodendrocyte-type 2 astrocyte (O2A) progenitor cells named OS3 was processed for western blot with (a) anti-LC3 antibody PM036 and relabeled with (b) anti-HSP60 antibody for standardization of protein loading. The lanes (1, 2) indicate a 48 hour-treatment of OS3 cells with (1) the equal v/v% concentration of dimethyl sulfoxide (DMSO) or (2) 1 μM rapamycin. [file 1750-1172-9-68-S1.ppt]

## Slide 1
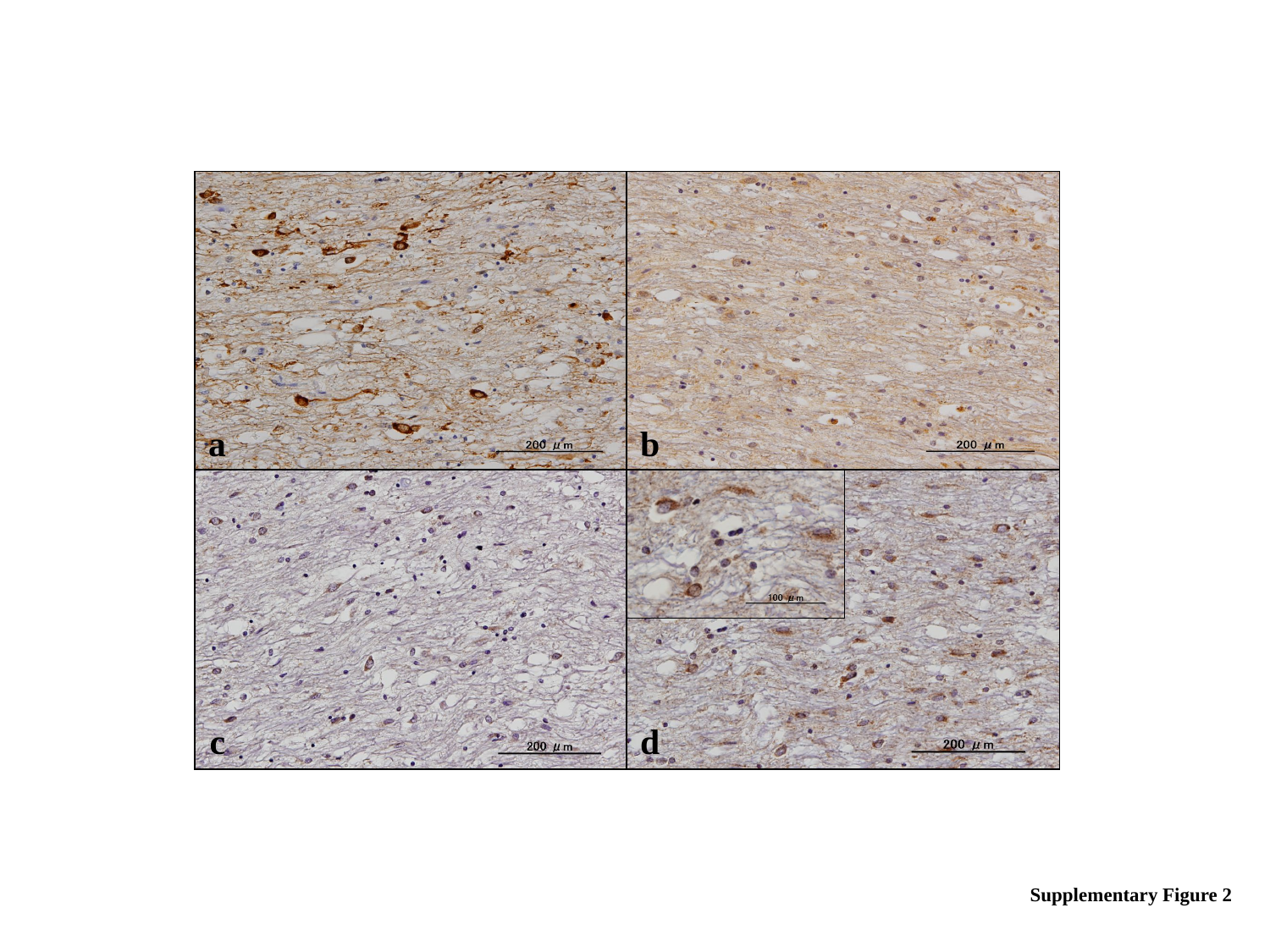

a
b
c
d
Supplementary Figure 2

Supplement: Additional file 2: Figure S2 — Surviving oligodendrocytes express HDAC6 in NHD brains. The serial brain sections of NHD cases were processed for immunohistochemistry. The panels (a-d) represent (a) the perivascular white matter, LC3, (b) the same field as (a), cleaved CASP3, (c) the same filed as (a), NBR1, and (d) the same field as (a), HDAC6 with a close-up view in inset. [file 1750-1172-9-68-S2.ppt]

## Slide 1
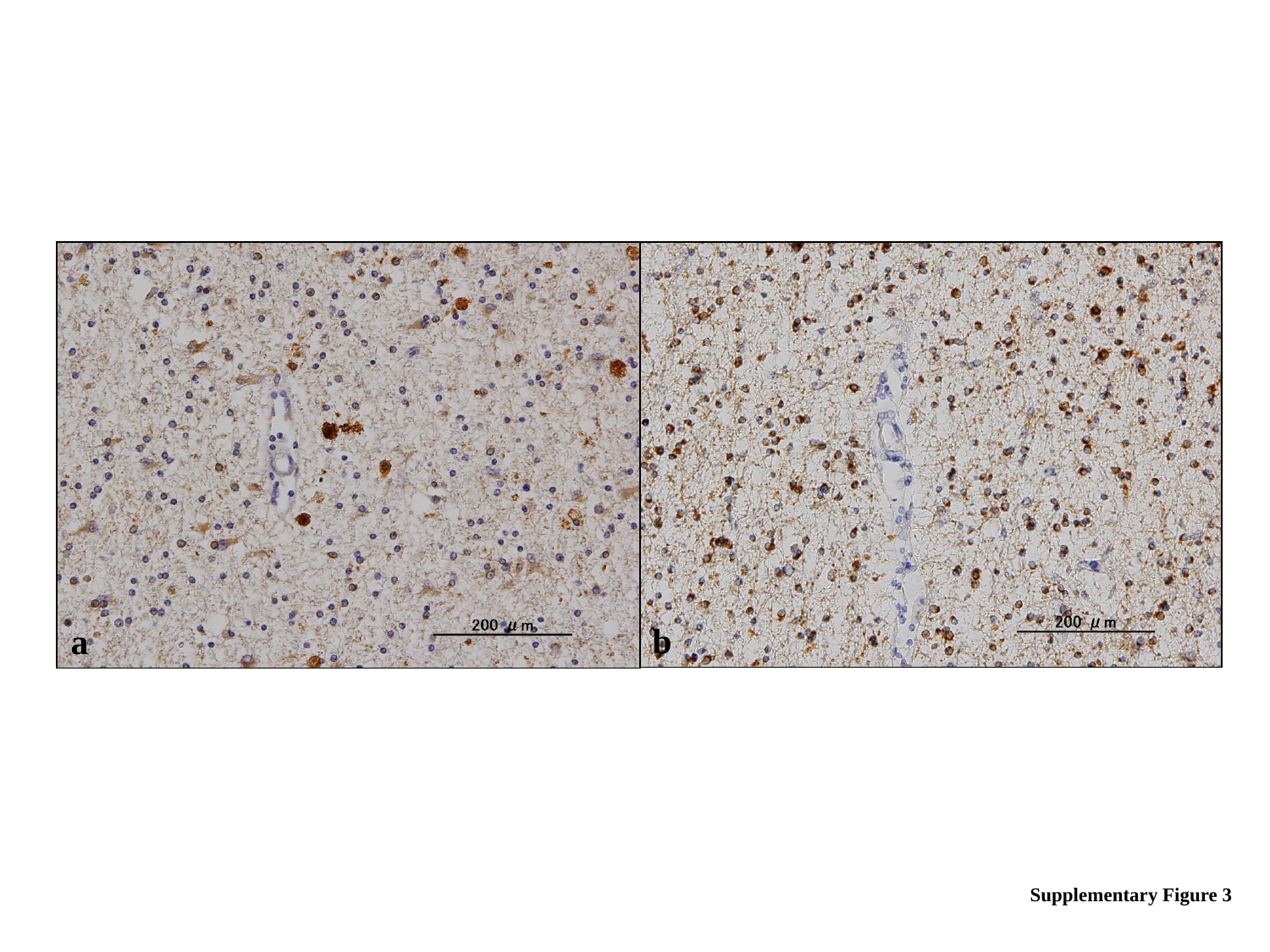

b
a
Supplementary Figure 3

Supplement: Additional file 3: Figure S3 — Oligodendrocytes do not express LC3 in early lesions of MS brains. The serial brain sections of MS cases were processed for immunohistochemistry. The panels (a, b) represent (a) an early lesion in the frontal white matter, LC3, some macrophages are positive, and (b) the same field as (a), Nogo-A. [file 1750-1172-9-68-S3.ppt]
